# Supplementary material for: Economic evaluation of point-of-care testing and treatment for sexually transmitted and genital infections in pregnancy in low- and middle-income countries: A systematic review
Source: PLoS One. 2021 Jun 17;16(6):e0253135. doi: 10.1371/journal.pone.0253135 (PMC8211269; doi:10.1371/journal.pone.0253135)
Supplement: Attachment — Submitted filename: PLOSONE_CEA POC testing_27Oct2020.docx [file pone.0253135.s003.docx]

Thank you for the opportunity to review this manuscript.

Overall assessment of originality and scientific quality

This manuscript provides the results of a systematic review of economic evaluations of antenatal point-of-care (PoC) testing and treatment for sexually transmitted infections (STIs) in low- and middle-income countries (LMICs). The authors identified 16 economic evaluations in the peer reviewed literature that met the inclusion criteria. Because of heterogeneity, authors were only able to provide a summary of the literature, and concluded that PoC testing and treatment during pregnancy is likely to be cost-effective compared to no testing, syndromic management, and lab-based testing. This is an important topic likely to be of interest to the readers of PLOS ONE. Most countries continue to provide sub-optimal antenatal STI care through the syndromic approach, missing infections and overtreating pregnant women. A major barrier to expanding etiological testing is cost and the uncertainty around the cost-effectiveness of testing. I suggest accepting this manuscript with some corrections and clarifications outlined below.

**Relevance to the field of this journal:**

The topic in general is relevant to the field and the topic is appropriate for publication in PLOS ONE. My suggestions for improvement, with examples from the text (with line numbers indicated) are presented below.

Presentation, tables and figures

- Readers should be able to understand the study objective in each of the figure and table titles. They should stand alone.

References

- Reference 8 is old. There are recent papers on the global burden of curable STIs
- Your only reference for adverse outcomes is for syphilis. Add in evidence for other STIs too.
- I think reference 18 should actually be 19.

Abstract, Introduction, Methods, Results and Discussion

***Abstract***

*Line 24*: “tests” and “facilitates” don’t agree (should be testing and facilitates or tests and facilitate).

*Line 26:* You mention “efficiency” often, but do you really mean effectiveness? What are your efficiency measures?

*Line 37*: should be “assessed”.

***Introduction***

Overall, your introduction is concise and comprehensive.

Paragraph 2 covers many ideas including: effectiveness of early detection and treatment of HIV and syphilis; interventions for managing STIs; syndromic management for CT/NG/TV; and lab-based diagnosis out of reach. I suggest breaking this paragraph up to have one paragraph on HIV & syphilis testing guidelines/protocols (e.g. types of testing conducted), and effectiveness; and another paragraph dedicated to the infections managed by syndromic management, problems with syndromic management and barriers to testing.

Paragraph 3 could benefit from the more description of point-of-care testing, such as desirable attributes (accuracy, minimal training, rapid, low cost, portability), etc.

Minor.

*Line 60*: When referring to the bacteria/protozoa for the first time, it would be good to spell out (e.g. *Chlamydia trachomatis*)

*Line 85*: All countries need to prioritize investment, but LMIC may have tighter budget constraints.

*Line 89*: Is there a citation for the new landscape that is emerging? What do you mean by this?

*Lines 100-105*: Are you not also comparing and contrasting effectiveness measures?

***Methods***

With respect to the literature review, I have several questions:

1) “Partial/ full economic evaluation” should be defined in the methods.
2) What software was used for the screening (e.g. Covidence)?
3) I have found that a lot of economic analysis has been conducted in the grey literature (e.g. by USAID/Health Policy Project, World Bank), why did you decide to exclude grey lit?
4) Does your search account for different spellings of diseases? (e.g. gonorrhea/ gonorrhoea/gonococcal)? What about prenatal (in addition to antenatal)?
5) Was the third reviewer/resolver more senior or have more expertise in the subject area?

With respect to the data abstraction process, I have several questions:

6) Did you abstract data associated with your quality appraisals (e.g. perspective, exchange rate)? Do you have a table that could be published in the supplemental documents?
7) What software was used for abstraction (e.g. Qualtrics/excel)?

***Results***

With respect to Table 3, I have several questions/comments:

1) I think it’s important to include the type of cost assessed in each study. As you note later, many studies did not include capital costs. Capital costs associated with POC testing often make this strategy unaffordable (compared to lab testing) because an assay must be placed at each clinical site.
2) It would be helpful to also add a column for effectiveness outcomes. If there’s no room, at least define what health outcomes are reflected in the DALYs (e.g. PID, preterm birth, conjunctivitis, etc.)

With respect to the results narrative, I have several questions/comments:

3) Please include more discussion on the details and quality of the outcomes included in the CEA/CUAs and separate out by syphilis/HIV and CT. Did authors use results from RCTs or meta-analyses? Were the effectiveness measures derived from an appropriate population – or are they all upper income populations? Was the intervention appropriate/similar? Do authors assume treated and cured infections have the same probability of an adverse outcome as never being infected?
4) In the quality assessment, do you consider how costs were collected (e.g. micro-costing / bottom up costing)?

*Line 226*: Does “treatment coverage” include loss to follow-up? I would think loss to follow-up would be important when comparing PoC with lab-based testing.

***Discussion***

1) The first paragraph isn’t really about your study. I would include this content in the introduction and use the first paragraph to summarize your most important findings.
2) Discuss drivers of costs/cost-effectiveness
3) Discuss the outcomes that were included. The effectiveness of CT/NG/TV infection testing and treatment in terms of preventing adverse health outcomes (besides PID) is very limited. This may be another important reason why countries are hesitant to introduce expensive testing.
   a. In the conclusion you mention a gap in terms of measuring the “life time costs and effects of testing and treatment on mothers and their babies,” but you don’t mention this in the discussion. What’s available in terms of lifetime costs of adverse outcomes? Do we need more research here?
4) Are the results similar to economic analyses in high income countries (e.g. Ong and Ditkowsky in Australia and the US)?

**Conclusion**

1) I disagree that your review “highlights the role that economic evidence plays in moving the global health agenda beyond the utility of antenatal point-of-care testing and treatment for STIs towards incorporating resource allocation, which is a major determinant of implementation and scale-up.” I think your review identifies and summarizes the literature, but more research is needed to move the agenda forward.
2) Again, mention cost/cost-effectiveness drivers.
3) Mention life time costs in the discussion if you are going to include in the conclusion.
