# Supplementary material for: Economic evaluation of point-of-care testing and treatment for sexually transmitted and genital infections in pregnancy in low- and middle-income countries: A systematic review
Source: PLoS One. 2021 Jun 17;16(6):e0253135. doi: 10.1371/journal.pone.0253135 (PMC8211269; doi:10.1371/journal.pone.0253135)
Supplement: Attachment — Submitted filename: SaweriOPM_Manuscript_Responses to editor.docx [file pone.0253135.s004.docx]

Editor-in-Chief

Plos one

Manuscript Title: Economic evaluation of point-of-care testing and treatment for sexually transmitted and genital infections in pregnancy in low- and middle-income countries: A systematic review

Authors: Olga PM Saweri, Neha Batura, Rabiah al Adawiyah, Louise Causer, William Pomat, Andrew J Vallely and Virginia Wiseman

On behalf of my co-authors, I would like to thank the editor and reviewers for your careful review of our manuscript. We greatly appreciate the time that you have generously given to this. We have revised the document according to the feedback given. Our responses are provided below.

Best,

Olga Saweri

**Reviewer #1 comments:**

Failing the option to do a formal statistical meta-analysis (due to heterogeneity in outcome measures and …), the crux of the findings are in the descriptive Table 3. In this table, however, between Results and outcome/conclusion the relation is not always clear, nor are the comparative outcome definitions under Results always clear. Please see Specific comments below.

RESPONSE: Thank you for your comment. The results and outcomes presented in Table 3 are results published by each study included in the review. Table 3 in the manuscript has been revised to clarify the relationship between the results and outcome/conclusion. The table now have 5 columns that present cost components, health outcomes (where applicable), efficiency measures, the drivers of cost effectiveness, and key findings of each economic evaluation. Each column has been systematically completed to improve coherence and comparability across the studies included in this review (see pages 9-17, line #190).

The discussion dwells on methods of economic evaluations and to what extend the existing, reviewed studies met or not the optimum standards. Apart from this partly academic matter, what is the more practical conclusion from the presented evidence? Concretely, if a health minister (in country X) considers changing its policy from syndromic treatment to point-of-care testing (for, say, syphilis), what could s/he learn from this review? Should a new (local?) study be carried out, and if so what would be the preferred methodology and design? Or is the evidence in favor of POC testing already clear and uncontested, in all or some (which=?) settings?

RESPONSE: Thank you for your comment, we have edited the discussion to explain a practical recommendation for implementation (see page 27-28, lines #354-369).

***Specific Comments:***

RESULTS: Table 3:

1) Shelley et al: What is meant with ‘Quality control measures valuable to scale-up of RST’? How does the ‘quality control’ relate to the Results? Please expand the Results such that this becomes clear. Also– if space allows – comment on how rural and urban settings differed, e.g. which setting had higher syphilis prevalence, or what was the driver of the comparative cost and screening and treatment volumes?

RESPONSE: The term ‘quality control’ is listed in column 8 of Table 3 and is now explained in the footnotes of Table 3 (denoted by *).

2) Bristow et al: What was the criterion for ‘best option’? I suppose, cost per DALY averted? If so, please add ‘cost per DALY averted’ to the results (possibly replacing cost and DALY). Or if you keep the outcome DALY: is this DALYs lost or DALYs averted?
   Adding an outcome per infection or DALY averted should help to make the study summary more comparable to other studies higher and lower within Table 3.

RESPONSE: This has now been made clearer through the inclusion of columns 8 and 9 of Table 3.

3) Owusu-Edusei et al: Same comment as for Bristow, on cost versus cost per DALY, and the definition if ‘more effective’, and of DALY (lost or averted).

RESPONSE: This has now been made clearer through the inclusion of columns 8 and 9 of Table 3.

4) Terris-Prestholt et al.: The ‘No screening program ; ICER reported as not applicable’ is redundant, as this is the definition of the counterfactual/comparator, ICERs are ex pressed against this counterfactual/comparator.

RESPONSE: The redundant statement has now been removed.

5) Kuznik et al: ICER is for what? Per infection averted? Please write that out. And clarify, what does an ICER mean for the ‘No screening program’, what was the comparator or counterfactual scenario here?

RESPONSE: This has now been clarified in Table 3 with a new column called ‘efficiency measures’ (column 9, row 10).

6) Rydzak: To interpret the ‘RST dominates’, please write out what is the comparator scenario for each scenario (No screening; RPR, and RST), for example: No screening compared to … RPR compared to No screening RST compared to ?RPR? or ?No screening?

RESPONSE: Table 3 has been revised and comparators for all full economic evaluations are detailed in column 5 of Table 3, while efficiency measures are now illustrated in column 10 of Table 3.

7) Schackman et al. Please clarify how Rural and Urban differed, e.g. in prevalence? Without that info, the differential results are meaningless to the reader.

RESPONSE: This has now been clarified in column 11, row 12 of Table 3.

8) Vickerman et al: From the 4 outcomes (cost/DALY) given, RPR has lower cost/DALY for Serum but RST has lower cost/DALY for Whole Blood. Therefore, the conclusion ‘test using serum/whole blood: Cost-effective outcome = RST’ is not obvious from the results – the results suggest RST as more cost-effective for whole blood but not for serum. Please clarify; for example, add into the Results cell, the corresponding cost per DALY for Serum + Whole Blood combined.

RESPONSE: The key result for this study has now been clarified in column 11, row 13 of Table 3.

9) Blandford: As for Kuznik et al., above, please clarify the respective comparators for each of the 3 testing options. Also clarify ‘Not published but states ‘Dominiated’.
   To facilitates readers’ matching between Conclusion and Results, I’d rephrase the Conclusion as ‘Off-site RPR and confirmatory TPHA’. And remove ‘(averts more cases)’ as that’s obvious from, and just a repeat of, the Results.

RESPONSE: Comparators for this study have now been added in column 5 and key study findings have been amended as requested and included in column 11.

10) Romoren et al: From the results, between the 4 options, the most cost effective looks to be Syndromic Management with azithromycin, at USD 21. In spite of that result, the conclusion states POC with azithromycin, despite it costing USD31 per cured case. Please reconciliate and clarify.

RESPONSE: This has now been clarified in column 11, row 17of Table 3. The results and the conclusion are now consistent.

11) Larson et al.: Where does the 62% come from, what algorithm or policy was this the result of?
    At reading the conclusion, I infer back that ‘RST’ must correspond to test and treat all, i.e. the third of the 3 options. Please write this out in the Results. And perhaps shorten the corresponding scenario names in column Intervention versus Comparator.

RESPONSE: The source of the 62% has now been added Footnote (denoted **) of Table 3). The link between the results and comparators have also been made clearer. The scenario names have also been made clearer. We have listed an explanation in the footnotes of Table 3 (denoted with **).

***Discussion:***
The Discussion mentions many wise and true things, but they are not too well structured, leaving the reader without a clear understanding of the overall current evidence or its policy implications.

We would recommend structuring the discussion of limitations between:
1) Limitations that affect the core outcomes of studies analyzed (in Table 3), i..e. that bias those comparisons, notably:

• Societal perspective, which may cause under-stating the value POC compared to laboratory-based policies, if the savings from POC are for a large part on the patient/client side.
• Types of cost: if the variability across studies is important, then please show it – adding a column with ‘Cost components included’ to Table 3. (To make space in Table 3, you might remove the column Intervention versus Comparator’ which gets repeated in the next-right column in any case.?)

2) More general limitations or qualifications such as:
• Most studies costed only test kits, treatment and staff, but not QAQC or procurement: if this applies across all scenarios compared within a study, that comparison is not biased.
• Affordability and equity: True but somewhat beyond the scope of this review?
• Short time horizons: A true and important limitation – but not one that biases the comparison across scenarios within a study: Per treatment or maternal infections cured, the longer-term effects should be similar regardless of through which screening algorithm that treatment or cure was produced, not?

RESPONSE: Thank you for your comment, the limitations have now been grouped as recommended (see page 28, lines #370-383).

***Conclusion***:
The start and end of the conclusion as written are more an Introduction. I suggest to narrow and focus the conclusion on the part in the middle, from ‘Our review indicates…’ to ‘… other screening practices’.

RESPONSE: Thank you for your comment, we have made changes to the Conclusion as recommended by the reviewer.

Regarding the lack of geographical representation of the studies:
Why is that a problem? Is there reason to think that the alternative screening algorithms would compare differently in different settings (e.g. in Latin America or Asia-Pacific)? Why, what setting-specific determinant (e.g. STI prevalence??) would be the driver of varying cost-effectiveness rankings? If you believe this is truly important, then make it more concrete, beyond a description of geographic representation, in the 3rd paragraph of the Discussion.

RESPONSE: A sentence has been added to the discussion emphasising the importance of conducting economic evaluations in a range of settings (see page 25, lines #292-295).

**Reviewer #2:**

***Presentation, tables and figures***

- Readers should be able to understand the study objective in each of the figure and table titles. They should stand alone.

RESPONSE: Thank you for your suggestion, we have renamed all the tables and the figures in this manuscript to reflect the study objective in each.

***References***

- Reference 8 is old. There are recent papers on the global burden of curable STIs
- Your only reference for adverse outcomes is for syphilis. Add in evidence for other STIs too.
- I think reference 18 should actually be 19.

RESPONSE: Thank you for your feedback; we have included recent references for chlamydia, gonorrhoea, trichomonas, and bacterial infection: REFS #1-REF#9 (see page 3, line #59). We have replaced reference #8 with a newer study: REF#14 (see page 3, line #62). We have also replaced reference #18 with two newer references: REF#26 and REF#27 (see page 3, line #75).

***Abstract, Introduction, Methods, Results and Discussion***

***Abstract***

*Line 24*: “tests” and “facilitates” don’t agree (should be testing and facilitates or tests and facilitate).

RESPONSE: This sentence has now been edited (see page 1, line #24).

*Line 26:* You mention “efficiency” often, but do you really mean effectiveness? What are your efficiency measures?

RESPONSE: Efficiency measures are now listed for all studies in column 10 of Table 3.

*Line 37*: should be “assessed”.

RESPONSE: This correction has been made (see page 1, line #38).

***Introduction***

Paragraph 2 covers many ideas including: effectiveness of early detection and treatment of HIV and syphilis; interventions for managing STIs; syndromic management for CT/NG/TV; and lab-based diagnosis out of reach. I suggest breaking this paragraph up to have one paragraph on HIV & syphilis testing guidelines/protocols (e.g. types of testing conducted), and effectiveness; and another paragraph dedicated to the infections managed by syndromic management, problems with syndromic management and barriers to testing.

RESPONSE: Thank you for your suggestion, we have amended the manuscript to reflect this suggestion (see page 3-4, lines #64-70 and lines #71-78).

Paragraph 3 could benefit from the more description of point-of-care testing, such as desirable attributes (accuracy, minimal training, rapid, low cost, portability), etc.

RESPONSE: Thank you for your suggestion. Desirable attributes for both serological and molecular point-of-care tests are described on page4 lines #83-85.

***Minor***

*Line 60*: When referring to the bacteria/protozoa for the first time, it would be good to spell out (e.g. *Chlamydia trachomatis*)

RESPONSE: Indeed, when referring to the causative organism (bacteria/protozoa) it is convention to list the name of the bacteria in full. However, for this manuscript, we are referring to, and identifying, the infection/disease (chlamydia) rather than the bacteria *(chlamydia trachomatis)*, therefore we have listed the infection in the manuscript.

*Line 85*: All countries need to prioritize investment, but LMIC may have tighter budget constraints.

RESPONSE: This edit has been made on page 5 (see lines #94- #95).

*Line 89*: Is there a citation for the new landscape that is emerging? What do you mean by this?

RESPONSE: “…new landscape emerging” refers to the recently developed and *effective* technology for testing and treatment at point-of-care. This has been clarified on page 5 (line 98) by the inclusion of supporting references for Chlamydia, Gonorrhoea, and HPV (REFS #37, #41, #44).

*Lines 100-105*: Are you not also comparing and contrasting effectiveness measures?

RESPONSE: Thank you for your feedback, the purpose of this review is to systematically review the evidence on economic evaluations of point-of-care testing to detect STIs in pregnancy. We are comparing and contrasting the primary outcome of these studies, which include costs and cost-effectiveness, not effectiveness measures per se and clarified this (see page 6, lines #111-112).

***Methods***

***With respect to the literature review, I have several questions:***

1) “Partial/ full economic evaluation” should be defined in the methods.

RESPONSE: Brief definitions of partial and full economic evaluations have been included in the methods section (see page 7, lines #139-144).

2) What software was used for the screening (e.g. Covidence)?

RESPONSE:

We have revised the manuscript to include the software package (Microsoft Excel (version 365)) used for screening records, titles and abstracts (see page 7 line #135).

3) I have found that a lot of economic analysis has been conducted in the grey literature (e.g. by USAID/Health Policy Project, World Bank), why did you decide to exclude grey lit?

RESPONSE:

We excluded grey literature as it is may not always follow recommended guidelines for conducting economic evaluations and/or is not often peer reviewed. By excluding grey literature, we appreciate that we may have excluded some relevant evidence and acknowledge this as a limitation (see page 28, lines #379-381).

4) Does your search account for different spellings of diseases? (e.g. gonorrhea/ gonorrhoea/gonococcal)? What about prenatal (in addition to antenatal)?

RESPONSE:

The search terms were finalised in consultations with a medical librarian. The terms and syntax used (Table 2) account for different spelling. For example, “GONORRHEA/” takes the American spelling of Gonorrhoea (i.e gonorrhea) into consideration and different derivations of “Gonorrhoea”. Prenatal is the parent term for antenatal, however upon closer inspection during the development of the search strategy, the exclusion of prenatal did not influence the outcome of the search.

5) Was the third reviewer/resolver more senior or have more expertise in the subject area?

RESPONSE:

Yes, the third reviewer is more senior, and is a more experienced economist specialising in economic evaluations in low- and middle- income countries (LMIC) and has published 3 other systematic reviews in high impact journals including *Lancet Infectious Diseases* and *Health Economics*.

***With respect to the data abstraction process, I have several questions:***

6) Did you abstract data associated with your quality appraisals (e.g. perspective, exchange rate)? Do you have a table that could be published in the supplemental documents?

RESPONSE: We have not abstracted the data for the quality appraisals. However, in response to the comments from reviewer 1 we amended Table 3, which now presents relevant details extracted from the studies included in this review and somewhat overlaps with the checklist items in the quality appraisal. We have also amended the manuscript to reflect this (see page 7, lines #152-156).

7) What software was used for abstraction (e.g. Qualtrics/excel)?

RESPONSE: Microsoft Excel (version 365) was used for abstraction. The manuscript has been revised to make this clearer (see page 7, line #152).

***Results***

***With respect to Table 3, I have several questions/comments:***

1) I think it’s important to include the type of cost assessed in each study. As you note later, many studies did not include capital costs. Capital costs associated with POC testing often make this strategy unaffordable (compared to lab testing) because an assay must be placed at each clinical site.

RESPONSE: Types of costs (Cost components) are now detailed in column #7 of Table 3.

2) It would be helpful to also add a column for effectiveness outcomes. If there’s no room, at least define what health outcomes are reflected in the DALYs (e.g. PID, preterm birth, conjunctivitis, etc.)

RESPONSE: In Table 3, the column entitled ‘efficiency measures’ incudes the primary measure of effectiveness used in the economic evaluation (see column #9).

***With respect to the results narrative, I have several questions/comments:***

1) Please include more discussion on the details and quality of the outcomes included in the CEA/CUAs and separate out by syphilis/HIV and CT. Did authors use results from RCTs or meta-analyses? Were the effectiveness measures derived from an appropriate population – or are they all upper income populations? Was the intervention appropriate/similar? Do authors assume treated and cured infections have the same probability of an adverse outcome as never being infected?

RESPONSE: we have amended the text to highlight the number of studies conducted using trial data and those conducted using existing literature (See page 18, lines #200-201).

2) In the quality assessment, do you consider how costs were collected (e.g. micro-costing / bottom up costing)?

RESPONSE: Thank you for your comment, we did not include this in our analysis. However, we know that only four studies (all of the partial economic evaluations) clearly stated that they utilised an ingredients-based approach, three of which also indicated utilising a step-down approach to costing. All of the full economic evaluations (n=12) did not specify a method for how costs were collected, we could only establish that these studies took an ingredients based approach, however, could not establish if another approach was also utilised.

*Line 226*: Does “treatment coverage” include loss to follow-up? I would think loss to follow-up would be important when comparing PoC with lab-based testing.

RESPONSE: The key drivers presented in this sub-section are those that are identified by the studies included in this review, we have only collated them to compare/contrast them. Treatment coverage refers to the number of antenatal clinic attendees treated for an STI. However, we cannot extrapolate whether treatment coverage includes loss to follow-up (unless specifically stated in each study).

***Discussion***

1) The first paragraph isn’t really about your study. I would include this content in the introduction and use the first paragraph to summarize your most important findings.

RESPONSE: Thank you for your suggestion, we have amended the first paragraph of the discussion as suggested (See page 25, lines #267- 282)

2) Discuss drivers of costs/cost-effectiveness

RESPONSE: Thank you for your comment, we have revised the manuscript to include a paragraph on the drivers of cost and cost-effectiveness (See page 26, lines #318- 325).

3) Discuss the outcomes that were included. The effectiveness of CT/NG/TV infection testing and treatment in terms of preventing adverse health outcomes (besides PID) is very limited. This may be another important reason why countries are hesitant to introduce expensive testing.

RESPONSE: Thank you for pointing this out, indeed there is limited evidence demonstrating the effectiveness of testing and treating CT/TV/NG in pregnancy to reduce adverse pregnancy and birth outcomes such as miscarriage, still births, neonatal deaths etc. However, as detection of CT/TV/NG has improved over the last decade, there has been a growing number of random control trials investigating the effectiveness of detecting CT/TV/NG in pregnancy to reduce adverse pregnancy and birth outcomes, enabling the expansion of evidence and providing a definitive conclusion whether testing and treating STIs in pregnancy reduces adverse pregnancy and birth outcomes (See page 26, lines #296-303).

4) In the conclusion you mention a gap in terms of measuring the “life time costs and effects of testing and treatment on mothers and their babies,” but you don’t mention this in the discussion. What’s available in terms of lifetime costs of adverse outcomes? Do we need more research here?

RESPONSE: In the discussion we mention that most economic evaluations are based on a relatively short time-horizon and overlook these long-term costs and effects (see page 26 lines #313- #317).

5) Are the results similar to economic analyses in high income countries (e.g. Ong and Ditkowsky in Australia and the US)?

RESPONSE: Cost-effectiveness studies for STIs, including that of Ong and Ditkowsky, conducted in high income countries tend to not focus on testing at point-of-care unless conducted in a remote community. Thus making it difficult to compare results of studies based in high-income countries.

***Conclusion***

1) I disagree that your review “highlights the role that economic evidence plays in moving the global health agenda beyond the utility of antenatal point-of-care testing and treatment for STIs towards incorporating resource allocation, which is a major determinant of implementation and scale-up.” I think your review identifies and summarizes the literature, but more research is needed to move the agenda forward.

RESPONSE: We have toned down this statement to these concerns (see pages 28-29).

2) Again, mention cost/cost-effectiveness drivers.

RESPONSE: We have revised the manuscript to include the ‘drivers of cost and cost effectiveness’ in the conclusion (see page 29, lines #388-391).

3) Mention life time costs in the discussion if you are going to include in the conclusion

RESPONSE: Please see previous response. These costs are now mentioned in the discussion (see page 26 lines #313- #317).
