# Supplementary material for: Economic evaluation of point-of-care testing and treatment for sexually transmitted and genital infections in pregnancy in low- and middle-income countries: A systematic review
Source: PLoS One. 2021 Jun 17;16(6):e0253135. doi: 10.1371/journal.pone.0253135 (PMC8211269; doi:10.1371/journal.pone.0253135)
Supplement: Attachment — Submitted filename: Peer review SAWERI Econ Point-of-care tests STI pregnancy LMIC_PLOSONE_25Jan2021rev1_ek1.docx [file pone.0253135.s005.docx]

**Peer review ‘Economic evaluation of point-of-care testing and treatment for STI and genital infections in pregnancy in LMIC: a systematic review – Revision 1**

By SAWERI-O, WISEMAN-V *et al.*

for PLoS ONE, 25 January 2021

RECOMMENDATION:

Minor revision

GENERAL/MAJOR COMMENTS

The authors have made revisions in response to each comment, which has strengthened the Methods, Results and Discussion – but I wonder if some of the changes are adequate, and some may actually introduce confusion or misinterpretation.

Table 3 is more readable now. Still, this being the key results, it merits further improvement – and an attempt to restore some meaningful content dropped since the original submission.

SPECIFIC COMMENTS

RESULTS, Table 3:

Drivers of cost and cost-effectiveness: This is a useful addition, in principle – but does not really yet add insight in practice, because:

- The drivers mentioned are much broader than those that determine the ranking between study arms, i.e. RST versus comparators, shown in the left-hand column.
- The drivers mentioned pertain mostly to the absolute cost per outcome (e.g. $$ per case averted or per DALY) but that column got dropped since the original submission.
- The cost drivers mentioned are named too broad and vague, for example: ‘Cost’ does this mean procurement cost i.e., test price?

I would suggest to be more concrete and specific in listing cost drivers -- focusing on the specific factors that explain the comparison between RST and comparators – and explain the direction of effect – which study arm/comparator had higher or lower value for each cost driver. Or optionally, slit the column into 2:

- Drivers of ‘absolute’ cost per outcome;
- Drivers of the difference in cost-per-outcome between RST and comparators.

You could make space by removing the column Infection studied, replacing that with one start or footnote indicating the one study where this was not syphilis but chlamydia. And some other of the left-hand columns might be merged.

For example, in Shelley et al., which of the 5 factors explained that RST rollout was cheaper than pilot? Which of the two used which type of blood collection? Which of the two arms had longer or shorter lifetime, and how did that influence the cost?

Sweeney et al.

- If the efficiency measure was total cost per health facility, did each health facility in both study arms test and treat equally many persons?
- What is meant with Time taken to test, is that the personnel time per test? Was that time longer for RST than for RPR?

Levin: In the column cost drivers, could you state the factors that explained the contrasting cost ranking between Mozambique and Bolivia? That does not seem to require the study having included a sensitivity analysis (?)

Owusu-Edusei et al: Was cost associated with adverse pregnancy outcome really a cost driver? Isn’t that relevant only if the incidence of such outcomes differed between the study arms i.e. comparators – which is unlikely: any undetected and untreated infection will on average incur the same adverse outcomes, regardless of the study arm /comparator that failed it? This is an example of my general comment above, that the many items mentioned in this column appear mostly meaningless. I suggest you limit the contents of this column to factors that influence the comparison between study arms / comparators. Any factors that influence the overall cost are not relevant, since your table (or any other Result in the paper) do not deal with the absolute overall cost or cost-effectiveness measures – they all just center on relative comparison of RST against comparators.

& Same comment for Rydzak.

Similarly, for Blandford, did the relative distribution of active/latent syphilis differ between the study arms i.e., comparators? If not, it is not relevant in this table that focuses on comparing test policies.

Kuznik 2015 & 2013, and Vickerman: Rephrase ‘All comparators’ as ‘Comparator’ since these studies each had only 1 comparator.

Terris-Prestholt et al.: As for Levin above, please focus and limit the cost drivers mentioned on those that explain the difference in ranking (RST above or below comparators) between Peru, and Tanzania+Zambia..

Kuznik 2013: By definition, the Discount rate will always influence the absolute cost measure, but it will do so equally for all study arms and comparators, and all studies not just Kuznik – so this is a meaningless redundant item to mention here.

Rydzak, Shackman, & Romoren:: Rephrase ‘All comparators’ as ‘Both comparator’ since these studies each had 2 comparators.

Rydzak: 1000 women is women tested, or treated?

Romoren et al: Under point 3. Remove the redundant words ‘Point-of-care testing and Azithromycin treatment vs.’

In the right-most cell of this row, clarify that PoC is RST, and replace ‘and’ by ‘with’ to clarify that this (combo of test type + treatment type) is 1 study arm not 2.

Larson: The added footnote (answering my earlier comment: Where does the 62% come from, what algorithm or policy was this the result of?) is helpful. But I’m still left with the question, why only 10% of positive cases were treated, and which 10% was this, was this random or by any characteristic or policy criteria?

& Cosmetic/formatting: Make Table 3 shorter and more readable by adapting the column widths according to their average content.

DISCUSSION

Page 36: Please explain the ‘Largest vs smallest percentage difference, 170% and 19% - between what? For which outcome is this, and which 4 of the studies listed in Table 3 provided the extremes?

Page 37: *‘In particular, the fundamentals of an economy and disease epidemiology..*’: This reads as poor-language generality, I don’t understand what specifically the author want to say. Please rephrase into something more concrete, or remove.

*‘This has, in turn, led to a comparatively new and emerging field…*’: I think the discussion could do without this perhaps exaggerated statement.

‘*Economic evaluations… should ideally… incorporate the long-term costs and benefits…’*: I am not convinced this is necessarily neede -- unless the longer-term costs and/or benefits differ between the intervention arms being compared? Which does not seem to be the case for POC STI tests. Please also see my related specific comments on various rows/study summaries in Table 3.

Page 40: *‘… the incorporation of extended cost-effectiveness analyses and budget impact analyses into cost-effective analyses*…: This reads like triple circular.

Additionally, most studies took a narrow view… short-run time horizon, although this does not influence the comparability between studies, it does highlight a short/coming of the studies included in this review. Consider to shorten and simplify – the time horizon was discussed already earlier in the Discussion.

*‘…therefore grey literature…*’: These last 10 last words are redundant in the sentence.

*‘Finally, a further enhancement … equity’*: This duplicates the mentioning of equity in line 403 above?
