# Supplementary material for: Economic evaluation of point-of-care testing and treatment for sexually transmitted and genital infections in pregnancy in low- and middle-income countries: A systematic review
Source: PLoS One. 2021 Jun 17;16(6):e0253135. doi: 10.1371/journal.pone.0253135 (PMC8211269; doi:10.1371/journal.pone.0253135)
Supplement: Attachment — Submitted filename: Saweri_ResponseToReviewers.docx [file pone.0253135.s006.docx]

Editor-in-Chief

Plos one

Manuscript Title: Economic evaluation of point-of-care testing and treatment for sexually transmitted and genital infections in pregnancy in low- and middle-income countries: A systematic review

Authors: Olga PM Saweri, Neha Batura, Rabiah al Adawiyah, Louise Causer, William Pomat, Andrew J Vallely and Virginia Wiseman

On behalf of my co-authors, I would like to thank the editor and reviewers for providing a detailed and extensive second review of our manuscript. We greatly appreciate the time and effort put into the feedback we have received. We have revised the manuscript and our responses to the reviewers’ feedback are provided below.

Best,

Olga Saweri

RESULTS, Table 3:

1. Drivers of cost and cost-effectiveness: This is a useful addition, in principle – but does not really yet add insight in practice, because:

- The drivers mentioned are much broader than those that determine the ranking between study arms, i.e. RST versus comparators, shown in the left-hand column.
- The drivers mentioned pertain mostly to the absolute cost per outcome (e.g. $$ per case averted or per DALY) but that column got dropped since the original submission.
- The cost drivers mentioned are named too broad and vague, for example: ‘Cost’ does this mean procurement cost i.e., test price?

RESPONSE:

Thank you for your feedback. The results presented in Tables 3 and 4 illustrate the results published in each included study and our interpretation of those results. The drivers of cost and cost-effectiveness, which were previously included in Table 3 are now presented in Table 4; these drivers are the results of sensitivity analyses conducted by each study included in this review (see Column D in Table 4 on pages 16-22). The revisions made to Column D in Table 4 (pages 16-22) reflect the results published by each study included in this review and are less ambiguous. In addition, we specifically list the drivers of cost and cost-effectiveness in the manuscript text and cross-reference the results presented in Table 4 (see page 24, lines 248-252).

2. I would suggest to be more concrete and specific in listing cost drivers -- focusing on the specific factors that explain the comparison between RST and comparators – and explain the direction of effect – which study arm/comparator had higher or lower value for each cost driver. Or optionally, slit the column into 2:

- Drivers of ‘absolute’ cost per outcome;
- Drivers of the difference in cost-per-outcome between RST and comparators.

RESPONSE:

Thank you for this suggestion. We have revised the manuscript to reflect this wherever possible. However, this was challenging. Few studies explicitly differentiated between drivers of absolute cost per outcome and those the explain the comparison between study arms or comparators. Most studies do not explicitly state that the results of the sensitivity analysis were drivers of cost-effectiveness, cost, effectiveness or between arms (see Table 4, column D, pages 18-24). Where the text is not explicit, we have assumed that the results of the sensitivity analysis are drivers of cost-effectiveness for full economic evaluations (n=12) or drivers of cost for partial economic evaluations (n=4).

3. You could make space by removing the column Infection studied, replacing that with one start or footnote indicating the one study where this was not syphilis but chlamydia. And some other of the left-hand columns might be merged.

RESPONSE:

We have amended Table 3 and included an additional table (Table 4) to improve the spaces in each column heading to improve readability (see Table 3 on pages 10-15 and Table 4 on pages 16-22).

4. For example, in Shelley et al., which of the 5 factors explained that RST rollout was cheaper than pilot? Which of the two used which type of blood collection? Which of the two arms had longer or shorter lifetime, and how did that influence the cost?

RESPONSE:

In this paper, the sensitivity analysis parameters were screening coverage, test kit price, supply wastage, project life, and type of blood collection. Blood collection here refers to the difference in collecting blood via a finger prick and via venipuncture. The former is utilized for RST while the latter for RPR. The results of the sensitivity analysis indicate that cost per person screened is most sensitive to changes in screening coverage, however changes in RST test kit and supply wastage also impact cost per person. These results are now reflected in table 4 column D (pp18-24).

In response to the query about the time horizon, the costs of both the pilot and the rollout were collected over the same amount of time, just two years apart. Further, the sensitivity analysis measured the effect of the project life, whereby costs in the rollout period were more sensitive to a change in the project life compared to the pilot period.

5. Sweeney et al.

- If the efficiency measure was total cost per health facility, did each health facility in both study arms test and treat equally many persons?
- What is meant with Time taken to test, is that the personnel time per test? Was that time longer for RST than for RPR?

RESPONSE

No, in this study, health facilities did not test and treat equal numbers of women in the study arms. In the RPR arm, over a 9-month period between 2007 and 2008, 838 antenatal clinic (ANC) attendees were tested for syphilis at 6 of the 9 selected health facilities. This is 17.8% of ANC attendees. Further 27% of women tested positive for syphilis and about 66% of those who tested positive were treated. The results state that no confirmatory tests were completed, therefore the true prevalence is unknown.

In the RST arm, over a similar time frame (in 2009-2010) at the 9 selected health facilities, 9372 ANC attendees were tested (87% of total ANC attendees); 10% of antenatal clinic attendees tested positive, of which 92% were treated.

With respect to ‘the time taken to test’, there is a difference between the time taken to conduct an Rapid Plasma Reagin (RPR) test for syphilis compared to a point-of-care test, or rapid syphilis test (RST). An RST is ‘quicker’, the study does not specifically state the difference in time. The sensitivity analysis showed that both RST and RPR costs were sensitive to staff time.

6. Levin: In the column cost drivers, could you state the factors that explained the contrasting cost ranking between Mozambique and Bolivia? That does not seem to require the study having included a sensitivity analysis (?)

RESPONSE: It is not clear from Levin et al (2007), therefore we have not amended the manuscript. What is clear from Levin et al (2007) is that direct comparisons between total costs in each country would not lead to “meaningful” results as the ‘project periods and the numbers of women screened are not directly comparable’ (see page S50/S54).

With respect to the rank of costs, costs related to testing accounted for 50% to 84% of costs in both countries. Looking into the breakdown of costs, ‘tests and other supplies’ accounted for the greatest share of costs in both countries. In Bolivia, clinic staff salaries associated with testing accounted for the second largest share of costs followed by start-up costs. However, in Mozambique, lab supplies accounted for the second largest share of costs followed by clinic staff salaries.

7. Owusu-Edusei et al: Was cost associated with adverse pregnancy outcome really a cost driver? Isn’t that relevant only if the incidence of such outcomes differed between the study arms i.e. comparators – which is unlikely: any undetected and untreated infection will on average incur the same adverse outcomes, regardless of the study arm /comparator that failed it? This is an example of my general comment above, that the many items mentioned in this column appear mostly meaningless. I suggest you limit the contents of this column to factors that influence the comparison between study arms / comparators. Any factors that influence the overall cost are not relevant, since your table (or any other Result in the paper) do not deal with the absolute overall cost or cost-effectiveness measures – they all just center on relative comparison of RST against comparators.

RESPONSE:

Thank you for your comment. Yes, cost associated with adverse pregnancy outcome was a driver of cost, thus we have not made any amendments to the manuscript. Owusu-Edusei et al (2011) identifies drivers in the text, which we have included in this table. Table 2 (see page 1001/1003) illustrates that the incidence of adverse outcomes differs per study arm (total adverse pregnancy outcomes are highest when there is no screening program (n=39) and lowest when screening using an RST (n=2)).

Excerpt from Owusu-Edusei et al (2011) p1000/1003: “…we varied select variables and examined the total expected cost keeping all other variables constant. As expected, the relative test performance, test costs, and costs associated with adverse pregnancy outcomes were all influential.” The text continues to describe how all scenarios were cost-saving and the extent to which they were.

8. Same comment for Rydzak.

RESPONSE:

Thank you for your feedback, an excerpt from Rydzak and Goldie (2008) page 780/784 indicates which variables were drivers, including the ‘costs associated with congenital syphilis’, therefore we have not made this change to the manuscript. The excerpt: “variations in test performance, test costs, and costs associated with congenital syphilis had the greatest effect on results. The choice between the 2 single-visit strategies was influenced by their comparative test sensitivity, and the cost of the ICS [rapid syphilis test] test kit, labour and supplies. A screening strategy was considered the preferred strategy if it achieved greater benefits at lower cost than all other strategies.”

Further, table 3 (page 779/784) indicates the number of adverse pregnancy outcomes and the number of adverse outcomes averted under each comparator. The table demonstrates that adverse pregnancy outcomes differ per comparator.

9. Similarly, for Blandford, did the relative distribution of active/latent syphilis differ between the study arms i.e., comparators? If not, it is not relevant in this table that focuses on comparing test policies.

RESPONSE:

Thank you for your comment; Blandford et al (2007) illustrates on that ‘model effectiveness of antenatal screening approaches are most affected by test sensitivity’ (see page S64/S66). The article also states that prevalence of maternal syphilis and the distribution of active and latent syphilis indirectly impact cost-effectiveness through costs. Given this, we have not changed the drivers stated in Table 4 (see page 20).

10. Kuznik 2015 & 2013, and Vickerman: Rephrase ‘All comparators’ as ‘Comparator’ since these studies each had only 1 comparator.

RESPONSE:

Thank you for your suggestion. We have made relevant changes used consistent language across all studies. The key findings column has now been placed in Table 4 (see Column E, Table 4 on pages 16-22).

11. Terris-Prestholt et al.: As for Levin above, please focus and limit the cost drivers mentioned on those that explain the difference in ranking (RST above or below comparators) between Peru, and Tanzania+Zambia.

RESPONSE:

Thank you for your comment, Terris-Prestholt er al (2015) states on page S76/S80 that the ‘variability in the DALYs averted for this algorithm is due to the variability of RST reactivity rate (or positivity rate)’ in addition, the study also highlights that variability in total cost is driven primarily by ANC attendance, fixed clinic costs and screening coverage. These have all been included as drivers of cost and cost-effectiveness. We have not included ‘true prevalence’, as this did not affect the ranking (See Row 11, Column D in Table 4 on page 19)

12. Kuznik 2013: By definition, the Discount rate will always influence the absolute cost measure, but it will do so equally for all study arms and comparators, and all studies not just Kuznik – so this is a meaningless redundant item to mention here.

RESPONSE:

Thank you for your feedback, we have amended Table 4 to reflect this (see Column D, Table 4 on pages 16-22).

13. Rydzak, Shackman, & Romoren:: Rephrase ‘All comparators’ as ‘Both comparator’ since these studies each had 2 comparators.

RESPONSE:

Thank you for your suggestion. We have made relevant changes to Table 4 and used consistent language across all studies (see Column D, Table 4 on pages 16-22).

14. Rydzak: 1000 women is women tested, or treated?

RESPONSE:

Rydzak and Goldie et al (2008) states that their outcome measure is ‘discounted costs saved per 1000 women’, which includes both testing and treatment for syphilis.

15. Romoren et al: Under point 3. Remove the redundant words ‘Point-of-care testing and Azithromycin treatment vs.’ In the right-most cell of this row, clarify that PoC is RST, and replace ‘and’ by ‘with’ to clarify that this (combo of test type + treatment type) is 1 study arm not 2.

RESPONSE:

Thank you for your comment. We have amended the manuscript to reflect that ‘testing for chlamydia using a point-of-care test followed by treating those testing positives for chlamydia with azithromycin’ was the most cost-effective outcome (see Row 18, Column E, Table 3 on pages 14 and Row 18, Column D, Table 4 on page 21).

We would like to clarify that the point-of-care test here refers to a point-of-care test for chlamydia, and therefore is not a rapid syphilis test (RST). We have amended Table 3 and 4 to state ‘point-of-care’ test.

16. Larson: The added footnote (answering my earlier comment: Where does the 62% come from, what algorithm or policy was this the result of?) is helpful. But I’m still left with the question, why only 10% of positive cases were treated, and which 10% was this, was this random or by any characteristic or policy criteria?

RESPONSE:

We have edited the contents of table 3 to improve clarity (see Row 19, Column E of Table 3) and the footnote of Table 3 (see pages 14-15).

Larson et al (2014) built three scenarios using the results of an evaluation study exploring rollout of point-of-care testing and treatment for syphilis in antenatal clinics (ANC). The evaluation found that 62% of ANC attendees were tested for syphilis. All women testing positive should have been treated, but only 10% were. For clarity, the scenarios from this study are outlined below:

Scenario 1 (baseline): the results of the evaluation study found that 62% of attendees were tested for syphilis, while only 10% of those that tested positive were treated. Therefore scenario 1 emulated this: 62% of antenatal clinic attendees tested for syphilis and 10% of test positives were treated.

Scenario 2: explored the scenario of treating all antenatal clinic attendees testing positive yet keeping the number of antenatal clinic attendees tested for syphilis (62%) constant. Thus scenario 2 becomes testing 62% of antenatal clinic attendees and treating all antenatal clinic attendees that test positive for syphilis.

Scenario 3: which could be seen as the ideal scenario, explores the possibility of testing all antenatal clinic attendees and treating all of whom test positive for syphilis.

17. Cosmetic/formatting: Make Table 3 shorter and more readable by adapting the column widths according to their average content.

RESPONSE:

Thank you for your comment. We have edited and adjusted Table 3 to make it more readable (pages 10-15).

DISCUSSION

1. Page 36: Please explain the ‘Largest vs smallest percentage difference, 170% and 19% - between what? For which outcome is this, and which 4 of the studies listed in Table 3 provided the extremes?

RESPONSE:

The percentage differences have been calculated per study to express the measured differences between comparators of each study included in this review. We have included percentage differences in Column G of Table 4 (see pages 16-22) to illustrate the difference between the baseline figure and the most cost-effective comparator of each study included in this review. In addition to percentage differences the cost-effectiveness decision rule has been included in Column C of Table 4 to reflect how each study determined cost-effectiveness. Together these findings illustrate between-study variability (see lines 285-293, page 30). In addition, we have edited the methods section to highlight percentage differences briefly and their inclusion in the results (see lines 163-168, pages 7-8).

2. Page 37: *‘**In particular, the fundamentals of an economy and disease epidemiology..*’: This reads as poor-language generality, I don’t understand what specifically the author want to say. Please rephrase into something more concrete, or remove.

RESPONSE:

Thank you for your feedback, we have amended the manuscript (see lines 307-311, pages 30-31).

3. *‘This has, in turn, led to a comparatively new and emerging field…*’: I think the discussion could do without this perhaps exaggerated statement.

RESPONSE:

Thank you for your comment, we have amended the manuscript (see lines 313-316, page 31).

4. ‘*Economic evaluations… should ideally… incorporate the long-term costs and benefits…’*: I am not convinced this is necessarily neede -- unless the longer-term costs and/or benefits differ between the intervention arms being compared? Which does not seem to be the case for POC STI tests. Please also see my related specific comments on various rows/study summaries in Table 3.

RESPONSE:

Thank you for your feedback, the effects of untreated sexually transmitted infections in pregnancy can extend after birth, both in women and in children. Estimates of long-term costs and benefits would help us understand whether the investment in such programs can have a positive impact on the health of mothers and babies later in life.

5. Page 40: *‘… the incorporation of extended cost-effectiveness analyses and budget impact analyses into cost-effective analyses*…: This reads like triple circular.

RESPONSE:

Thank you for your suggestion, we have amended the manuscript to sound less ‘like [a] triple circular’ (see lines 379-382, page 33).

6. Additionally, most studies took a narrow view… short-run time horizon, although this does not influence the comparability between studies, it does highlight a short/coming of the studies included in this review. Consider to shorten and simplify – the time horizon was discussed already earlier in the Discussion.

RESPONSE:

We have shortened the sentence as suggested (see lines 394-395, page 33).

7. *‘**…therefore grey literature…*’: These last 10 last words are redundant in the sentence.

RESPONSE:

We have removed this sentence from the manuscript (see line 398, page 33).

8. *‘**Finally, a further enhancement … equity’*: This duplicates the mentioning of equity in line 403 above?

RESPONSE:

The previous round of reviewer comments suggested that the final paragraph of the discussion should be a ‘summary of the discussion’. Thus, the final sentence of this paragraph seems repetitious as it is a summary point of the discussion. We have amended the manuscript to sound less repetitious (see line 399-400, page 33).
